# Ectopic corticotropin-releasing hormone (CRH) syndrome from metastatic small cell carcinoma: a case report and review of the literature

**DOI:** 10.1186/1746-1596-5-56

**Published:** 2010-08-31

**Authors:** Sadeka Shahani, Rodolfo J Nudelman, Ramaswami Nalini, Han-Seob Kim, Susan L Samson

**Affiliations:** 1 Division of Diabetes, Endocrinology and Metabolism, Department of Medicine, Baylor College of Medicine, One Baylor Plaza, Houston, Texas 77030, USA; 2 Department of Pathology, Baylor College of Medicine, One Baylor Plaza, Houston, Texas 77030, USA; 3 Ben Taub General Hospital, 1504 Taub Loop, Houston, Texas 77030, USA

## Abstract

**Background:**

Cushing's Syndrome (CS) which is caused by isolated Corticotropin-releasing hormone (CRH) production, rather than adrenocorticotropin (ACTH) production, is extremely rare.

**Methods:**

We describe the clinical presentation, course, laboratory values and pathologic findings of a patient with isolated ectopic CRH causing CS. We review the literature of the types of tumors associated with this unusual syndrome and the behavior of these tumors by endocrine testing.

**Results:**

A 56 year old woman presented with clinical and laboratory features consistent with ACTH-dependent CS. Pituitary imaging was normal and cortisol did not suppress with a high dose dexamethasone test, consistent with a diagnosis of ectopic ACTH. CT imaging did not reveal any discrete lung lesions but there were mediastinal and abdominal lymphadenopathy and multiple liver lesions suspicious for metastatic disease. Laboratory testing was positive for elevated serum carcinoembryonic antigen and the neuroendocrine marker chromogranin A. Serum markers of carcinoid, medullary thyroid carcinoma, and pheochromocytoma were in the normal range. Because the primary tumor could not be identified by imaging, biopsy of the presumed metastatic liver lesions was performed. Immunohistochemistry was consistent with a neuroendocrine tumor, specifically small cell carcinoma. Immunostaining for ACTH was negative but was strongly positive for CRH and laboratory testing revealed a plasma CRH of 10 pg/ml (normal 0 to 10 pg/ml) which should have been suppressed in the presence of high cortisol.

**Conclusions:**

This case illustrates the importance of considering the ectopic production of CRH in the differential diagnosis for presentations of ACTH-dependent Cushing's Syndrome.

## Introduction

Cushing's syndrome (CS) comprises the clinical manifestations of exposure to elevated glucocorticoid hormones, either from exogenous sources or endogenous overproduction by the adrenal glands [1]. CS from endogenous production is rare with an incidence of 0.7 to 2.5 per million per year depending on the geographic region and population studied [2]. Although autonomous cortical adrenal adenomas or carcinomas are a cause of elevated cortisol, most cases (85 to 90%) are adrenocorticotropic hormone (ACTH) dependent and are caused by corticotroph pituitary adenomas (Cushing's Disease or CD) [1]. The remaining ACTH-dependent cases mostly involve non-pituitary malignancies (Ectopic ACTH) which often are tumors of neuroendocrine origin such as small cell lung carcinoma or bronchial carcinoid [3,4]. Here, we describe a case of Cushing's syndrome which initially appeared to be caused by ectopic ACTH secretion from metastatic small cell carcinoma. However, immunohistochemistry of the tumor cells was negative for ACTH expression, while corticotrophin releasing hormone (CRH) was strongly expressed, suggesting that the elevated ACTH levels were a result of pituitary secretion in response to CRH.

## Case report

A 56 year old type 2 diabetic patient presented to the emergency room with shortness of breath. Review of systems revealed back pain for two weeks, without history of injury, and progressive generalized weakness and fatigue for many months. She also described constant right upper quadrant abdominal pain. On examination, there was hyperpigmentation of her face and neck in sun exposed areas, thin skin with violacious striae on her lower abdomen, and multiple ecchymoses over the extremities, abdomen and at venipuncture sites. There was prominent fat accumulation in the supraclavicular and dorsocervical regions. There also was objective evidence of proximal muscle weakness which, along with back pain, had caused her to be bed-ridden for two weeks. Her medications included an oral sulfonylurea for diabetes but no exogenous sources of corticosteroids. Laboratory results revealed hypokalemic alkalosis (K^+ ^2.1 mEq/l and HCO_3 _42 mEq/l). She was hyperglycemic (random glucose 339 mg/dl). An MRI of the spine showed multiple endplate compression fractures of the thoracic and lumbar vertebrae.

A clinical diagnosis of CS was suggested by the presentation. The laboratory results are summarized in Table 1. Morning serum cortisol was extremely elevated and did not suppress after 1 mg Dexamethasone. The urinary cortisol was 16,575 μg/24 hours (normal 0-50 μg), which was confirmed on dilution. ACTH was elevated (278 pg/ml) in spite of hypercortisolism, confirming ACTH-dependent CS. A 48 hour high dose dexamethasone suppression test (2 mg every 6 hours for 8 doses) did not cause suppression of the serum cortisol suggesting that this was a case of ectopic ACTH and, in agreement, the pituitary gland had a normal appearance on MRI. Computed tomography (CT) of the chest did not reveal any lung nodules but did show mediastinal lymphadenopathy. CT abdomen showed multiple lesions in the liver and enlarged para-aortic and retroperitoneal lymph nodes suggestive of metastatic disease. The adrenal glands were prominent but were without focal nodularity.

**Table 1:** Laboratory Presentation

AT PRESENTATION (Normal Range)	VALUE
Serum Potassium (3.8-5.2 mEq/l)	2.1

Serum HCO3 (21-32 mEq/l)	42

Plasma Glucose (mg/dl)	339

Morning Serum Cortisol (06:15) (4.0-32 μg/dl)	111.1

**ENDOCRINE WORK-UP (Normal Range)**	

1 mg Overnight Dexamethasone Supression Test (Normal cortisol < 1.8 μg/dl)	116.7

48 hour High Dose Dexamethasone Supression Test (Normal cortisol < 1.8 μg/dl)	109.8

24 hour Urine Free Cortisol (0 to 50 μg)	16 575

Plasma ACTH (10-46 pg/ml)	278

Plasma CRH^a ^(0-10 pg/ml)	10

Plasma Chromogranin A (0-5.0 nmol/l)	26

Serum Carcinoembryonic antigen (0-5.0 ng/ml)	10.59

Serum Calcitonin (0-5 pg/ml)	< 2.0

Serum Serotonin (0-420 ng/ml)	156

Plasma Metanephrine (0-62 pg/ml) and Normetanephrine (0-145 pg/ml)	12 37

Serum Gastrin (13.0-115.0 pg/ml)	280

24 hr Urinary 5-HIAA (0-8.0 mg)	6.7

Abbreviations: ACTH, Adrenocorticotropic Hormone; CRH, Corticotropin Releasing Hormone, 5-HIAA, 5-hydroxy-indoleacetic acid

^a^CRH assay is a plasma based test by which Corticotropin Releasing Factor is measured by direct radioimmunoassay (Inter Science Institute, Inglewood, CA).

Because no primary lesion was found on imaging, a number of follow-up laboratory tests were performed. Chromogranin A and Carcinoembryonic antigen (CEA) were elevated consistent with a malignancy with neuroendocrine characteristics (Table 1). Laboratory tests for known causes of ectopic ACTH-namely, carcinoid (serotonin, 24 hour urine 5-hydroxy-indoleacteic acid), pheochromocytoma (plasma free metanephrines and normetanephrines), and medullary thyroid carcinoma (calcitonin)- were in the normal range (Table 1). Gastrin was over 2-fold higher than the upper limit of normal.

With the exception of the liver metastases and enlarged lymph nodes, there were no discrete lung nodules or neck, abdomen or pelvis lesions seen on CT. Scintigraphy with [^111^-In]pentreotide showed no tracer uptake at 4 hours. Specimens from bronchoalveolar lavage did not show any histologic findings of small cell lung carcinoma.

**Figure 1:**
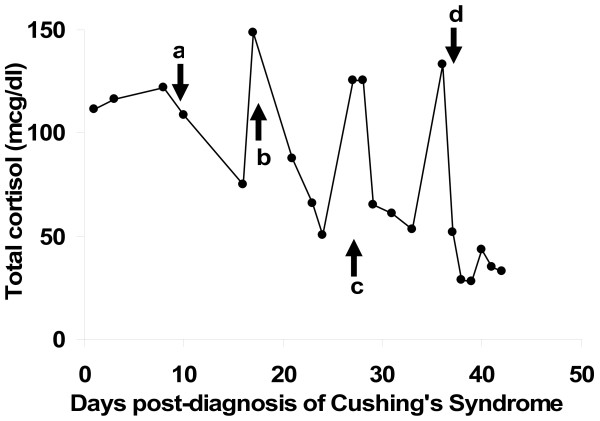
**Plasma cortisol levels during the patient's hospital course and treatment.** a = ketoconazole 200 mg PO BID, b= ketoconazole 400 mg BID, c= ketoconazole 400mg TID, d= metyrapone 500 mg QID.

The patient's course continued to deteriorate, with hypokalemia, hyperglycemia and difficult to control blood pressure. She was started on ketoconazole, an inhibitor of cortisol synthesis, but her cortisol levels continued to escape inhibition after each dose escalation (Figure 1). The patient then was switched to metyrapone, resulting in a significant decrease in cortisol levels (Figure 1). Chemotherapy was initiated (cisplatin and vincristine) but the patient become hemodynamically unstable after one round of therapy and was transferred to the intensive care unit where she passed away. An autopsy was declined by the family which prevented confirmation of the origin of the small cell carcinoma.

## Pathological findings

**Figure 2:**
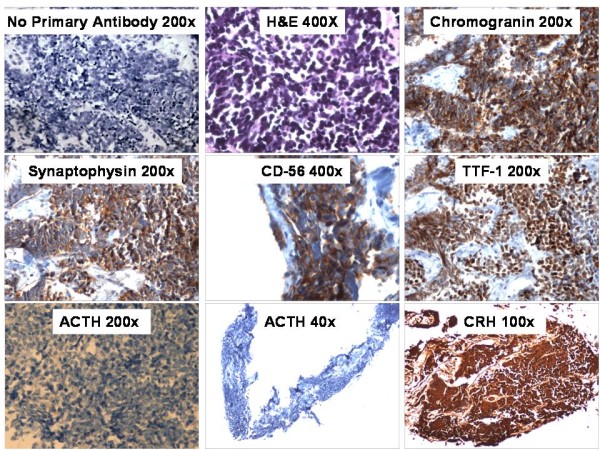
**Immunohistochemistry of biopsy samples from a CT guided biopsy of a liver metastasis.** The cells stained positively for chromogranin, synaptophysin, thyroid transcription factor 1 (TTF-1) and CD-56.  The cells stained negatively for ACTH but were strongly positive for CRH.

CT guided biopsy of one of the liver lesions was performed to obtain tissue for diagnosis. The biopsy consisted of multiple cores of soft tissue with no liver parenchyma present, although rare glandular structures were seen, suggestive of the residual bile ducts in the portal triad. On hematoxylin and eosin staining, there was extensive infiltration by small blue tumor cells surrounded by slender fibrous tissue (Figure 2). The cells were discohesive with small nucleoli, powdery chromatin, and no observable cytoplasm. The tumor cells were positive for neuroendocrine markers chromogranin, and synaptophysin as well as CD56 and Thyroid Transcription Factor-1 (TTF-1) (Figure 2). The cells were negative for the epithelial markers pancytokeratin and LC8 (CD45). These histopathologic findings were suggestive of small cell carcinoma. Surprisingly, immunostaining was negative for ACTH (Figure 2) (rabbit polyclonal antibody from Cell Marque, 1:200 dilution). Because of this, immunostaining for CRH was performed (rabbit polyclonal antibody from Sigma-Aldrich at a 1:200 dilution) which was strongly positive (Figure 2). Subsequently, a laboratory test for plasma CRH revealed that it was 10 pg/ml, at the upper limit of normal (0-10 pg/ml), in spite of concurrent hypercortisolism (Table 1).

## Discussion

Cushing's syndrome (CS) is the clinical manifestations of cortisol overproduction from ACTH-independent (adrenal cortical adenoma or carcinoma) or ACTH-dependent mechanisms. Most ACTH-dependent CS is due to corticotroph pituitary adenomas (90%), while ectopic ACTH production only comprises around 10% of cases [4]. A significant proportion of ACTH-secreting neoplasms (7-19%) remain occult even after extensive investigation [4-6]. There have been few reports of ACTH producing tumors which co-secrete CRH and even fewer which describe ectopic CRH production in the absence of ACTH, as we suspect in this case. Overall, an extensive search of the literature revealed isolated CRH reported in 20 cases [7-17]. Medullary thyroid carcinoma (33%) and pheochromocytoma (19%) are the most prevalent among the cases of isolated ectopic-CRH, while carcinoid (5%) and small cell lung carcinoma (9.5%) are less common, different from ectopic ACTH cases, with the latter two as the most prevalent causes (Table 2)[7-17]. Interestingly, isolated CRH production from prostate cancer comprises 14% of the cases [9,14] and yet it is an extremely rare source of ectopic ACTH (1-3%).There are single reports of sellar choristoma and gangliocytoma with isolated CRH production [9,14].

**Table 2:** CRH Positive and ACTH negative Tumors causing Cushing's Syndrome

NEOPLASM	% of CASES (n = 21)	REFERENCES
Medullary Thyroid Cancer	33	[8,9,11,15,18]

Pheochromocytoma	19	[10,11,13,16]

Prostate Cancer	14	[9,17]

Small Cell Lung Carcinoma	9.5	[14,17]

Small Cell Carcinoma (occult primary)^1^	9.5	[14]

Carcinoid	5	[14]

Other	< 10	[14,9]

^1 ^Including the case presented here.

We believe our patient had a CRH secreting tumor due to strongly positive CRH staining, negative ACTH staining, and detectable plasma CRH. A simple explanation for the lack of ACTH immunostaining is that it is not expressed in the tumor cells. The polyclonal antibody used for our ACTH immunostaining also reacts with the parent propeptide, pro-opiomelanocortin, so that aberrant prohormone processing of POMC to ACTH is not an explanation. We have not examined whether POMC transcripts were present in the biopsy sample, but there is precedence that POMC RNA can be present without detectable pro-peptide or ACTH in the tissue [7,18]. In these cases, it is not clear whether the POMC transcripts are not properly translated, or the ACTH is secreted too soon after production to be stored [9]. In our patient, we only biopsied the metastatic lesions in the liver, so that we also cannot rule out that other non-hepatic metastatic lesions were able to produce ACTH.

An interesting aspect of this case is the lack of significant cortisol suppression by the high dose dexamethasone suppression test (HDDST; 2 mg dexamethasone every 6 hours for 48 hours), with baseline cortisol at 122.2 μg/dl before the test and 109 μg/dl after 48 hours. With isolated ectopic CRH, it is expected that CS develops through the stimulation of pituitary ACTH secretion with resultant adrenal cortisol production. Because of this, ACTH and cortisol levels potentially could suppress with HDDST, similar to a pituitary adenoma. Young and colleagues [19] reported this false positive suppression in a case of ectopic CRH-ACTH production, which also behaved like Cushing's disease by CRH stimulation testing and inferior petrosal sinus sampling (IPSS). However, in the case presented here as well as that of seven other ectopic CRH cases, the lack of suppression of ACTH and cortisol are consistent ectopic ACTH production, avoiding the superfluous performance of other, sometimes invasive, pituitary-centered procedures (e.g. IPSS) [3]. Similarly, a single dose of 8 mg dexamethasone (overnight) has not suppressed cortisol in cases of isolated ectopic CRH [12,17]. These findings confirm that the positive drive by CRH can overwhelm the negative feedback by elevated corticosteroid. The systemic CRH levels in the case reported here were at the upper end of normal. Most other published cases of ectopic CRH reports do not have the CRH levels available for comparison.

## Conclusion

We present a rare case of isolated ectopic CRH production from metastatic small cell carcinoma of unknown primary. From the literature, the tumors most commonly associated with ectopic CRH production are medullary thyroid carcinoma, pheochromocytoma, and prostate cancer. With ectopic CRH syndrome, it is assumed that the pituitary is the source of ACTH with secretion driven by CRH overproduction. However, these tumors have behavior distinct from pituitary corticotroph adenomas, and the majority appears to lack suppression with high dose dexamethasone. Although the clinical presentation may be similar to ectopic ACTH syndrome, it is important to have ectopic CRH syndrome in the differential diagnosis for Cushing's syndrome.

## Consent

The patient was deceased and attempts to contact the next of kin were not successful. Because of this, the case content was submitted to the Institutional Review Board and was determined to be exempt from IRB review.

## Competing interests

The authors declare that they have no competing interests.

## Authors' contributions

All of the authors have read and approved the final manuscript. SS wrote the initial draft of the manuscript and analyzed the clinical data. HSK and RJN performed the immunohistochemistry on the biopsy samples and provided interpretation of the pathological findings. SLS coordinated the manuscript, edited the entire draft, performed the CRH immunostaining, and wrote the discussion of the case. RN edited the entire draft for final submission.

## Author details

Department of Medicine, Division of Diabetes, Endocrinology, and Metabolism and Department of Pathology, One Baylor Plaza, Mail Stop 285, Baylor College of Medicine, Houston, TX, 77030.
